# Le bébé collodion: aspects cliniques et intérêt du diagnostic anténatal

**DOI:** 10.11604/pamj.2017.26.118.10025

**Published:** 2017-03-02

**Authors:** Ridha Fatnassi, Nédia Marouen, Houcem Ragmoun, Latifa Marzougui, Sabra Hammami

**Affiliations:** 1 Service de Gynécologie-Obstétrique, Hôpital Ibn Eljazzar, 3140, Kairouan, Tunisie

**Keywords:** Ichtyose, bébé collodion, dermatose congénitale, Ichthyosis, collodion baby, prenatal diagnosis, congenital dermatosis

## Abstract

Le bébé collodion est une forme sévère de l’ichtyose congénitale à révélation néonatale. Le tableau clinique est souvent caractéristique. L’évolution, quand elle n’est pas fatale, se fait le plus souvent vers l’ichtyose sèche. Grâce aux techniques de biologie moléculaire, le diagnostic prénatal est rendu possible dès la 10-12^ème^ semaine d’aménorrhée, permettant un conseil génétique. Quant au pronostic de la maladie, il dépend de plusieurs paramètres, à savoir le degré de l’atteinte initiale, la durée de la desquamation, ainsi que l’ichtyose sous-jacente. A l’occasion d’une nouvelle observation d’un bébé collodion issu à 34 SA, d’une parturiente ayant un cas index, et a évolution fatale dés le premier jour de vie; nous avons fait une revue de la littérature afin de préciser les aspects diagnostiques et thérapeutiques ainsi que l’utilité du diagnostic anténatale.

## Abstract (en)

Collodion baby is a severe form of congenital ichthyosis detected in neonatal period. It often has a characteristic clinical picture. When evolution is not fatal; it often causes dry Ichthyosis. Thanks to molecular biology techniques, prenatal diagnosis can be made since the 10-12 weeks of amenorrhea, allowing genetic counselling. Prognosis depends on several parameters, namely the degree of the initial manifestation, the duration of desquamation, as well as underlying Ichthyosis This rewiew of the literature which aims to clarify the diagnostic aspects and therapeutic treatment as well as the role of the antenatal diagnosis is based on a new observation of a collodion baby born at 34 weeks, of a parturient woman having an index case and of infant deaths occurring in the first day of life.

## Introduction

L’ichtyose congénitale type bébé collodion est une maladie génétique cutanée rare [1]. Son nom vient du grec « ichthys » signifiant « poisson » et faisant référence à l’apparence clinique d’une peau écailleuse. Elle provoque une hyperkératinisation de l’épiderme. La peau devient alors épaisse et dure, avec de profondes fissures à sa surface [2]. Cette expression initiale est commune aux différents troubles de la différentiation épidermique et peut être observée dans différents types d’ichtyoses C’est le mode de début préférentiel des grandes ichtyoses transmises en récessivité [3]. L’affection est due à la rétention d’une couche cornée anormale in utéro. En effet, le processus pathologique mis en cause affecte la formation, le maintien et la fonction de la couche cornée. C’est donc « un trouble de la cornification » [4]. Le pronostic de cette affection dépend de la prise en charge dans la période néonatale. C’est dire la nécessité d’un diagnostic précoce et d’une prise en charge adéquate pour optimaliser ce pronostic.

## Patient et observation

**Figure 1:**
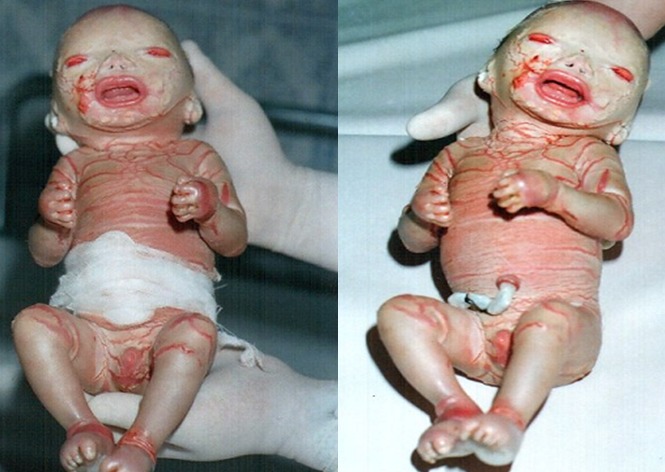
Aspect morphlogique d’un bébé collodion à la naissance

M.A, âgée de 27 ans, sans antécédents pathologiques notables, ayant comme antécédents gynéco-obstétricaux un avortement tardif au quatrième mois et un accouchement prématuré à 28 SA d’un bébé pesant 800g et présentant des lésions dermatologiques généralisées rappelant le bébé collodion. Elle nous a consultés pour des douleurs pelviennes à type de contractions utérines sur une grossesse de 33SA. A l’examen clinique avait noté la présence de contractions utérines régulières et douloureuses, une hauteur utérine à 27 cm et au toucher vaginal: un col raccourci, ouvert à un doigt avec une poche des eaux bombantes. L’échographie obstétricale avait montré une grossesse mono-fœtale évolutive dont la biométrie est conforme au terme. Par ailleurs, aucune anomalie morphologique n’avait été décelée à cet examen. Le bilan pré-tocolytique n‘avait révèle aucune anomalie. Une corticothérapie anténatale à base de Céléstène, ainsi qu’une tocolyse avec du Bricanyl par voie parentérale avaient été instaurées. L’évolution était marquée par un accouchement prématuré dans les 24 h qui suivent d’un bébé de sexe masculin, de poids de naissance 1600g, présentant une peau tendue et brillante L’évolution était marquée par un accouchement prématuré dans les 24 h qui suivent d’un bébé de sexe masculin, de poids de naissance 1600g, présentant une peau tendue et brillante ressemblant à du collodion avec un ectropion, une éversion des lèvres, des pavillons auriculaires fripés et des extrémités des doigts effilées (Figure 1). Son évolution était rapidement fatale dans les heures qui suivent. L’examen fœto-pathologique avait conclut à un fœtus collodion. Il s’agit donc d’un cas d’ichtyose congénitale récidivante.

## Discussion

Les ichtyoses sont un groupe hétérogène de maladies ayant pour caractéristiques communes la formation d’une couche cornée anormale avec des lésions cutanées hyperkératosiques qui se traduisent par une desquamation généralisée avec, ou sans, hyper prolifération épidermique ou inflammation du derme [1]. Le bébé collodion est l’expression congénitale et initiale de nombreuses formes d’ichtyoses; mais il ne permet pas de préjuger de la sévérité. Le nouveau né présente une peau semblable à du collodion desséché recouvrant toute la surface épidermique. L’exfoliation commence tôt par des craquelures de la membrane collodionnée. Les craquelures peuvent rester superficielles, ou s’approfondir et toucher le derme superficiel réalisant alors des fissures [2]. La peau érythémateuse, luisante, tendue et vernissée est responsable d’un ectropion, d’un éclabion, d’un hélix aplati et d’une flexion des doigts et des orteils. Les plis sont fissurés, parfois érosifs. Les semi muqueuses, les ongles et les cheveux sont normaux. Dans notre observation, les lésions dermatologiques étaient assez caractéristiques de l’affection, ce qui nous a permis de porter le diagnostic dès l’examen clinique. Le syndrome du bébé collodion doit être différencié des simples hyperkératoses collodionnées de la post maturité, du syndrome du Christ-Siemens-Touraine ou dysplasie ectodermique hypohidrotique liée à l’X, ainsi que du kératome malin (fœtus harlequin) : qui est la plus grave forme d’ichtyose connue, dans laquelle le fœtus apparaît recouvert d’une « carapace » rigide et fissurée et dont l’évolution est le plus souvent fatale dans les premiers jours de vie [3]. Le diagnostic positif du bébé collodion, ainsi que les diagnostics différentiels, sont avant tout cliniques. En cas de doute, l’étude de la biopsie cutanée confirmera l’hyperkératose orthokératosique. La récurrence de ce syndrome au sein d’une famille jusqu’au là indemne nous oriente vers une atteinte génétique transmise selon un mode autosomique récessif. La pathogénie en est mal connue. Certaines spécificités socioculturelles sont à l’origine d’un fort taux de consanguinité et d’une fréquence plus élevée de ces troubles héréditaires de la kératinisation.

Par ailleurs, une minorité des bébés collodions, associés à un déficit en transglutaminase kératinocytaire (TGK) par mutation de la transglutaminase 1, sont spontanément résolutifs [5]. En effet, cette variante suit une transmission autosomique récessive et un cas causé par une mutation particulière avec déficit à minima de transglutaminase kératinocytaire a été rapporté [3]. L’évolution est très variable et imprévisible. En effet, un tableau congénital de bébé collodion a été rapporté dans 53% des érythrodermies congénitales sèches et dans 12% des ichtyoses lamellaires. Dans 10% des cas, le bébé collodion peut être le premier signe d’une ichtyose vulgaire. Dans 10% des cas, il s’agit d’un self healing bébé collodion, alors que dans 20% des cas, il peut s’agir d’une maladie de Gaucher ou d’une Trichothiodystrophie montrant à la naissance un phénotype de bébé collodion [6, 7]. Le bébé collodion est un fœtus à haut risque. Il nait souvent prématurément [1], comme l’illustre notre cas.

L’étude de l’activité enzymatique de la TGK sur culture ou par immunofluorescence sur coupe de peau et la recherche de mutation du gène de la TGK peuvent être effectuées précocement permettant un diagnostic rapide et une consultation de conseil génétique. En effet, la gravité de cette forme d’ichtyose congénitale justifie le diagnostic prénatal, possible par réaction PCR génomique sur matériel des villosités choriales et la nécessité du conseil génétique [3, 8]. Le diagnostic anténatal peut être réalisé, s’il existe un antécédent d’ichtyose grave dans la famille, soit par fœtoscopie et biopsie cutanée fœtale à partir de la vingtième semaine d’aménorrhée, mais au mieux ce diagnostic prénatal est possible dès la 10-12^ème^ semaine de grossesse par PCR génomique sur matériel des villosités du chorion [1]. Ce diagnostic moléculaire est, alors, préféré permettant un diagnostic précoce et un retentissement psychologique moindre. Lequel diagnostic anténatal sera proposé aux familles comportant au moins un membre atteint. Une consultation de conseil génétique permettra d’informer les parents et de proposer une stratégie de diagnostic prénatal lors des grossesses ultérieures. Le pronostic du bébé collodion dépend de plusieurs paramètres. Ainsi, à la période néonatale, il est fonction des complications liées l’état du bébé collodion, et plus particulièrement aux complications hydro-électrolytiques, infectieuses et respiratoires. Alors qu’à long terme, il dépend du cadre dans lequel s’inscrit le bébé collodion. La prise en charge de ces nouveaux nés en milieu de réanimation néonatale a permis d’abaisser le taux de mortalité à moins de 10%. Cette mortalité est passée de 33% en 1976 à 11% en 1984 [3].

Dans un tiers des cas, la maladie est fatale suite aux infections ou à des désordres métaboliques [9]. L’essentiel du traitement est symptomatique. Il vise à réduire l’hyperkératose et à contrôler les possibles complications tant à la période néonatale qu’ultérieurement. Ce traitement repose sur l’hydratation, la lubrification (émollients) et la kératolyse (les kératolytiques). Le clinicien ne limitera pas son action à l’atteinte cutanée, mais s’attachera à prévenir les conséquences (fonctionnelles, sensorielles et psychologiques) pouvant être très affichantes pour l’avenir de ces enfants. La prise en charge doit être spécialisée. Elle englobe toute la famille, avec une consultation de génétique comportant une information aux possibilités actuelles de diagnostic anténatal pour les grossesses ultérieures.

## Conclusion

La naissance d’un bébé collodion représente une prise en charge lourde pour la famille et la société. Son dépistage anténatal est une question critique. Le décryptage génétique des diverses variétés d’ichtyoses progresse sur un rythme effréné. Un diagnostic spécifique sur un individu ou une famille avec ichtyose congénitale aide à établir un pronostic qui est fondamental pour un conseil génétique.
